# Items of General Interest

**Published:** 1915-05

**Authors:** 


### Items of General Interest.

We have just received the forty-first report of the Cincinnati Sanitarium, along with the notice of a new building which has been erected especially for the purpose of treating nervous patients. This new building, called "Rest Cottage," which will be ready for occupancy about July 1st, is modern in every respect, and is the last word in completeness. The report which is intensely interesting, showing a vast amount of work as having been done by the Sanitarium in the last year, is illustrated with very handsome cuts, showing both the exterior and interior of the Cincinnati Sanitarium. The personnel of the medical staff is as follows: F. W. Langdon, M. D., Medical Director; Drs. B. A. Williams and E. A. North, Resident Physicians; Dr. Georgie E. Finley, Matron, and H. P. Collins, President and Business Manager.

What is known as the "D. D. D." Lunch Club was organized by the doctors, dentists and druggists of Dallas on March 29th at the Oriental Hotel, for the purpose of instilling harmony between the men of the profession and for recreation purposes. The following officers were elected: Dr. H. B. Decherd, President; Drs. J. E. Simmons and Z. E. Marvin, First Vice-Presidents, and Dr. H. L. Whitaker, Secretary and Treasurer.

The JOURNAL is in receipt of the program of the State Medical Association, which convenes in Fort Worth, May 4-6. It is intensely interesting, and we regret that limited space prevents its publication.

Some twenty-five or thirty of the most prominent medical journals in the United States will give space in their July issue to what is known as the "Cancer Campaign." This JOURNAL will be one of the number to contribute space to this great work.

Dr. Beverly T. Young, formerly with the State Board of Health, but now a physician of San Antonio, was elected Superintendent of the Southwestern Insane Asylum April 2d, to succeed Dr. F. S. White.

In the course of a week or two work will be begun on what is known as the "Cleburne Sanitarium." This building will be modern in every respect and will be under the direct supervision of Dr. T. S. Jackson, formerly Superintendent of the Des Moines, Iowa, Sanitarium.

Major General William C. Gorgas, Surgeon General of the

United States Army, has been invited by the Rockefeller Foundation to become a permanent member of its staff in the capacity of general adviser in matters relating to public sanitation and the control of epidemics. General Gorgas is wanted by the foundation particularly at this time to direct the campaign against the typhus scourge which is devastating Serbia. General Gorgas has taken the invitation under consideration, but it is understood that he expects to consult Secretary of War Garrison and possibly President Wilson before reaching a decision.

Dr. Ernest P. Magruder, of Washington, D. C., one of the physicians at the head of the American Red Cross unit in Serbia, has fallen a victim to typhus fever. His death was reported on the 9th instant from Belgrade.

Dr. Alph H. Boehmer, for five years surgeon general of Siam and private physician to the late King Chulalongkorn, who has returned to this country, declares that he can cure leprosy and that there is little danger of contracting the disease. Dr. Boehmer intends to go to Cuba to study the treatment of tropical diseases.

Notice of a case which outrivaled that of the Siamese twins occurred in the *Houston Chronicle* recently. Two little girls of Spanish decent are bound together by a band of flesh which is 13 inches in circumference. The parents are carrying the children to the Drs. Mayo, who feel that they shall be able to separate them. We shall await with interest the outcome of the effort. We believe if the operation can be done successfully the Mayos can do it.

